# Mesenchymal stem cells for cardiac repair: are the actors ready for the clinical scenario?

**DOI:** 10.1186/s13287-017-0695-y

**Published:** 2017-10-27

**Authors:** Santiago Roura, Carolina Gálvez-Montón, Clémentine Mirabel, Joaquim Vives, Antoni Bayes-Genis

**Affiliations:** 1 ICREC Research Program, Germans Trias i Pujol Health Research Institute, Badalona, Spain; 2 Center of Regenerative Medicine in Barcelona, Barcelona, Spain; 3 CIBERCV, Instituto de Salud Carlos III, Madrid, Spain; 4 Servei de Teràpia Cel∙lular, Banc de Sang i Teixits, Edifici Dr. Frederic Duran i Jordà, Passeig Taulat, 116, 08005 Barcelona, Spain; 5 Musculoskeletal Tissue Engineering Group, Vall d’Hebron Research Institute, Universitat Autònoma de Barcelona, Passeig de la Vall d’Hebron 129-139, 08035 Barcelona, Spain; 6 Department of Medicine, Universitat Autònoma de Barcelona, Barcelona, Spain; 7 Cardiology Service, Hospital Universitari Germans Trias i Pujol, Badalona, Barcelona, Spain; 8 ICREC (Heart Failure and Cardiac Regeneration) Research Programme, Health Sciences Research Institute Germans Trias i Pujol (IGTP), Carretera de Can Ruti, Camí de les Escoles s/n, 08916 Badalona, Barcelona, Spain; 9 Heart Institute, Hospital Universitari Germans Trias i Pujol University Hospital, Carretera de Canyet s/n, 08916 Badalona, Barcelona, Spain

**Keywords:** Cardiac adipose tissue, Cardiac repair, Cell therapy, Good manufacturing practice, Mesenchymal stem cells, Tissue engineering, Umbilical cord blood

## Abstract

For years, sufficient progress has been made in treating heart failure following myocardial infarction; however, the social and economic burdens and the costs to world health systems remain high. Moreover, treatment advances have not resolved the underlying problem of functional heart tissue loss. In this field of research, for years we have actively explored innovative biotherapies for cardiac repair. Here, we present a general, critical overview of our experience in using mesenchymal stem cells, derived from cardiac adipose tissue and umbilical cord blood, in a variety of cell therapy and tissue engineering approaches. We also include the latest advances and future challenges, including good manufacturing practice and regulatory issues. Finally, we evaluate whether recent approaches hold potential for reliable translation to clinical trials.

## Background

Heart failure is a common, incapacitating, and ultimately deadly malady. It represents an important contributor to the enormous economic costs and loss of lives worldwide each year [1]. Currently, sufficient progress has been made in treatments that alleviate symptoms and reasonably prolong the lifespan. However, these treatments are palliative in nature; they do not address the underlying problem of functional heart tissue loss. In this field of research over the last 15 years, the research laboratory Insuficiència Cardíaca i Regeneració Cardíaca (ICREC; translated from Catalan: Heart Failure and Cardiac Regeneration) has actively explored innovative biotherapies for cardiac repair [2].

**Fig. 1:**
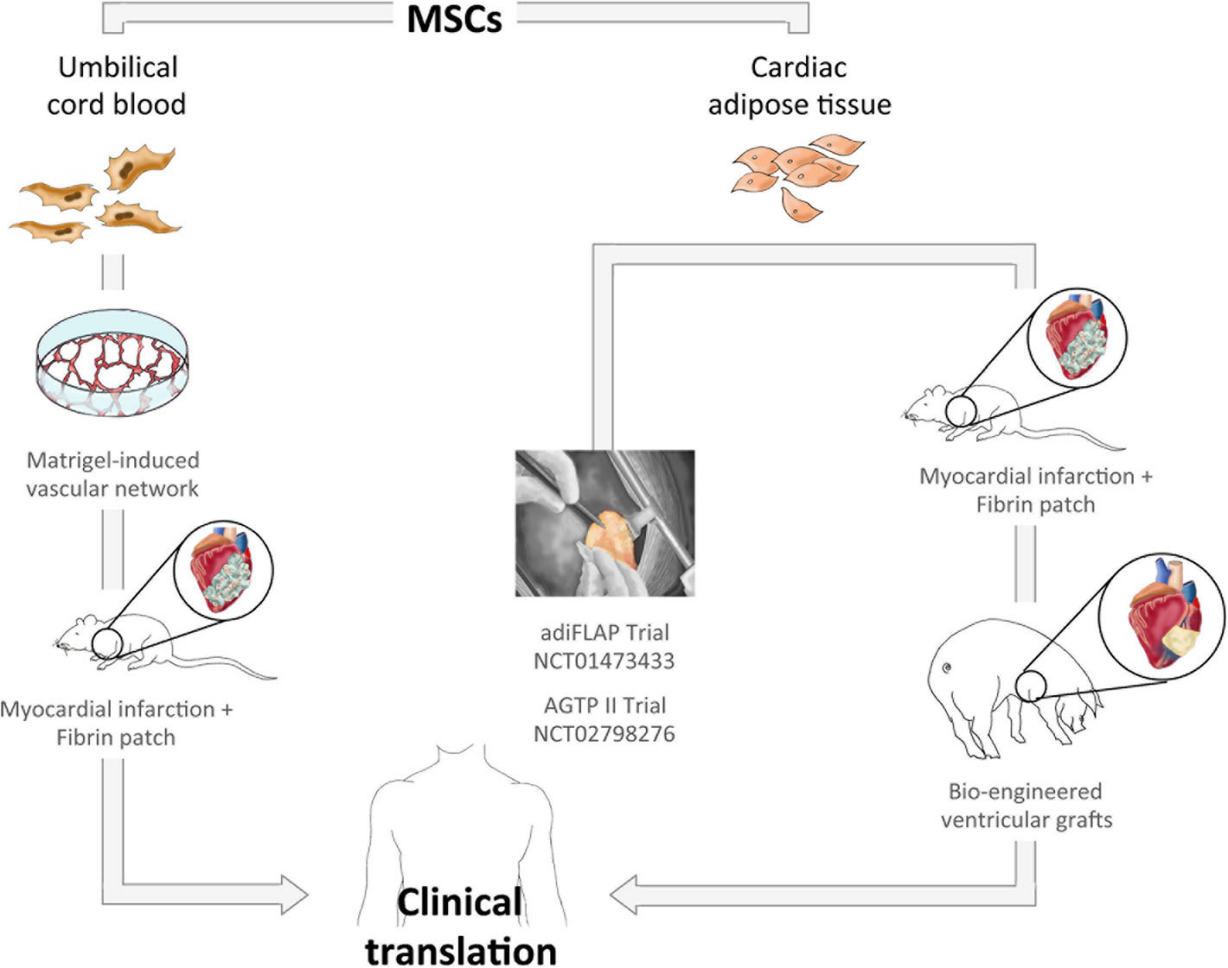
Cardiac cell therapy and tissue engineering experience at the ICREC laboratory. In the preclinical setting, UCBMSCs emerged as an alternative tissue source for cell therapy in treating diseases with vascular deficits. Conversely, cATMSCs showed baseline cardiomyogenic traits, which could be promoted with electromechanical stimulation; cATMSCs also possessed great reparative potential following implantation in vivo with cardiac engineered grafts or bioprostheses. Preliminary results were good when the AGTP was tested in humans; therefore, it might be not necessary to isolate and manipulate cells ex vivo when treating heart failure after myocardial infarction. At this time, our cell therapy and tissue engineering approaches are ready to undergo the complex good manufacturing practice and regulatory procedures necessary for translation into clinical scenarios. AdiFLAP pericardial adipose pedicle, AGTP adipose graft transposition procedure, MSC mesenchymal stem cell

In the following, we describe our work from a critical perspective with the aim of providing value. In particular, we review our experience over the years in addressing cardiac healing after myocardial infarction (MI). We have used a variety of mesenchymal stem cells (MSCs), including those derived from cardiac adipose tissue (cATMSCs) and umbilical cord blood (UCBMSCs), and cardiac repair strategies, including cell therapy, electromechanical stem cell conditioning, tissue engineering (TE), and an adipose graft transposition procedure (AGTP) (Fig. 1 and Table 1).

**Table 1:** Cell therapy and tissue engineering studies from the ICREC laboratory on UCBMSCs, cATMSCs, and the AGTP

Study	MSC	Model	Delivery method	Results	Biosecurity
Prat-Vidal et al. (2007) [33]	UCBMSC	In vitro	–	Isolation and characterization	–
Roura et al. (2010) [34]	UCBMSC	In vitro	–	No cardiomyogenic differentiation	–
Roura et al. (2012) [28, 36]	UCBMSC	Acute MI mice	Fibrin patch	Reduced infarct size, larger vessels in myocardial area, and improved cardiac function	No mortality
Bayes-Genis et al. (2010) [22]	cATMSC	Acute MI mice and rat	Intramyocardial	Cardiomyogenic and endothelial differentiation, improved cardiac function, reduced infarct size, increased vessel density	No mortality, No teratoma formation
Perea-Gil et al. (2015) [27]	cATMSC	In vitro	–	Dose-dependent suppression of T-cell alloproliferation	–
Llucià-Valldeperas et al. (2015) [39]	cATMSC	In vitro	–	Higher constitutive expression of Cx43, α-actinin, SERCA2, and GATA4	–
Llucià-Valldeperas et al. (2017) [40]	cATMSC	Acute MI mice	Fibrin patch	Improved cardiac function, increased myocardial vessel density	No mortality
Gálvez-Montón et al. (2011) [58]	cATMSC	Acute MI swine	AGTP	Improved cardiac function, reduced infarct size, flap-myocardium vascular connections	No mortality
Gálvez-Montón et al. (2013) [59]	cATMSC	Chronic MI swine	AGTP	Reduced infarct size, flap-myocardium vascular connections	No mortality
Prat-Vidal et al. (2014) [53]	cATMSC	Acute MI swine	Pericardial scaffold	Reduced infarct size	No mortality
Bayes-Genis et al. (2016) [62]	cATMSC	Chronic MI human	AGTP	Partial efficacy	No mortality
Perea-Gil et al. (2016) [56]	cATMSC	Acute MI swine	Myocardial scaffold	Improved cardiac function, reduced infarct size, less fibrosis, higher myocardial vessel density	No mortality, no rejection, no arrhythmias
Gálvez-Montón et al. (2017) [60]	cATMSC	Acute MI swine	Pericardial scaffold	Improved cardiac function, reduced infarct size, less inflammation and fibrosis	No mortality

*ICREC* Insuficiència Cardíaca i Regeneració Cardíaca, *UCBMSC* umbilical cord blood mesenchymal stem cell, *cATMSC* cardiac adipose tissue mesenchymal stem cell, *MI* myocardial infarction, *AGTP* adipose graft transposition procedure

## Evidence-based rationale for changing an old dogma: the rise of cell therapy

Over much of the last century, within the cardiac field, the mammalian adult heart was thought to be terminally differentiated. However, many studies have shown that myocardial regeneration occurred in rodents (1960s) [3], amphibians (1974) [4, 5], and zebrafish (2002) [6]. Subsequently, the human heart became a focus of intense research in regenerative medicine, due to its incapacity for self-repair. However, crucial studies have shown that resident cardiac stem cells maintained myocardial homeostasis throughout life [7–9], cardiomyocyte cell cycle activity continued to function at low levels [10–13], and cardiac chimerism/microchimerism phenomena occurred when extracardiac progenitor cells were moved to the myocardium, as in gender-mismatched heart transplantation procedures [14–19]; these observations have instigated a divergence from the old dogma. Hence, currently, we know that the human adult heart has reparative potential; unfortunately, however, this potential is reduced after MI, due to a massive loss of cardiac muscle. This condition causes an overload in the surviving myocardium and, potentially, leads to heart failure [20]. In present times, the only treatment for advanced heart failure that fully restores cardiac function is a heart transplantation, which is often restricted by the shortage of donors. These events have given rise to cell-based therapies.

Multipotent MSCs appeared on stage as an attractive option for regenerating damaged tissues. Briefly, MSCs are recognized for their ability to differentiate into osteogenic, chondrogenic, and adipogenic lineages in vitro, their typical fibroblast-like morphology, their adherence to plastic, when maintained in standard culture conditions, and their nonhematopoietic cell surface pattern. In general, evidence has suggested that the contribution of MSCs to MI recovery lies in paracrine signaling, rather than a direct effect of MSCs. Paracrine signaling would be consistent with findings that a low number of retained or seeded cells could promote restorative effects, such as forming vessels to protect resident cardiomyocytes from apoptosis and mobilizing resident stem cells to potentiate vascularization and cardiomyogenesis. Thus, some authors have proposed the term “medicinal signaling cells” to reflect the fact that MSCs integrate into the sites of injury and secrete immunomodulatory and trophic factors, which have either pharmacological or tissue reparative activities [21].

## cATMSCs: a source of stem cells with great cardiac potential

The trajectory of our laboratory has focused on the adipose tissue surrounding the heart as a source of MSCs [22]. In particular, we showed that these cATMSCs could be extracted from adipose depots located at the base of the heart and around the aortic root, from patients who underwent cardiothoracic surgery prior to cardiopulmonary bypass surgery. We hypothesized that cATMSCs, which showed a MSC-like pattern of cell surface antigen expression, might play a role in heart homeostasis, perhaps as a cell reservoir for renewing myocardial tissue. Indeed, despite residing in an adipose environment, cATMSCs had an inherent cardiac-like phenotype. At the protein level, they expressed β-MHC, SERCA2, sarcomeric α-actinin, GATA4, Cx43, and traces of Tbx5. In addition, coculturing cATMSCs with neonatal rat cardiomyocytes qualitatively increased the expression levels of these specific cardiac markers, and induced de-novo expression of troponin I, an important sarcomeric protein that was not observed in unstimulated cultures. Unexpectedly, unlike genuine MSCs, culturing cATMSCs in adipogenic differentiation medium did not result in the intracellular accumulation of lipid droplets. Other baseline traits of this novel cell population included that they were clonogenic and had a cell duplication time of about 5 days.

Interestingly, cATMSCs were also shown to preserve the myocardium, when delivered intramyocardially in postinfarcted mice and rats [22]. The engrafted cells expressed cardiac (troponin I, sarcomeric α-actinin) and endothelial (CD31) markers, and their administration was associated with enhanced myocardial vascularization and reductions in infarct size. Moreover, in terms of cardiac function, significant differences between control and cell-treated groups were found in fractional shortening and the ejection fraction; moreover, in the treated group, the anterior walls of the heart remained significantly thicker 30 days after cardiac delivery of cATMSCs compared to those of the control group.

Together with their recognized regenerative potential, MSCs have been linked to immune regulation through modulation of monocyte polarization toward an anti-inflammatory phenotype [23, 24] and interference in dendritic cell maturation [25]. For instance, Wharton’s jelly-derived MSCs powerfully inhibit the inflammatory response of stimulated T cells [26]. In this way, cATMSCs also abrogated T-cell proliferation upon stimulation with allogeneic mature monocyte-derived dendritic cells [27]. In a coculture setting, similar to the well-established nonimmunogenic UCBMSCs, increasing amounts of cATMSCs suppressed the alloproliferation of T cells in a dose-dependent manner, and specifically modulated secretion of proinflammatory cytokines (IL-6, TNF-α, and IFN-γ). Of note, these findings suggest that cATMSCs could regulate a potentially harmful immune response, despite the reported short lifespan of MSCs after infusion in vivo.

## UCBMSCs: a source of stem cells with great vascular potential

UCB is considered the most plentiful reservoir of stem cells for many clinical applications. Although UCB was commonly used to treat blood disorders, the spectrum of diseases for which it provides effective therapy has been expanded to include numerous nonhematopoietic conditions [28–30]. Consequently, the number of blood services/UCB banks established worldwide has continued to grow to accumulate sufficient supplies of donated units to meet the demands of cell transplantation. In addition to hematopoietic progenitor cells, UCB also contains nonhematopoietic cell types that can be readily isolated and grown ex vivo. MSCs represent one of the additional cell populations found in UCB. In short, UCBMSCs comprise a population of multipotent progenitor cells that can support hematopoiesis in bone marrow niches, differentiate into mesenchymal cell lineages (i.e., osteogenic, adipogenic, and chondrogenic lines), and display immune modulatory activity [31, 32]. Cell growth assays performed in our laboratory showed that UCBMSC proliferation, measured as the cell duplication time, was close to 2 days [33].

In past years, studies have exposed UCBMSCs to a myriad of cardiomyogenic stimuli and failed to achieve transdifferentiation to a cardiac lineage [34]. Nevertheless, through those studies, UCBMSCs have garnered a great deal of attention, which stimulated studies on the molecular mechanisms involved in regulating angiogenesis [35], the induction of vascular growth in vivo [36], and methods for preclinically predicting the immunogenicity of prospective stem cells [27]. For instance, although direct contact with neonatal rat cardiomyocytes could effectively induce a cardiomyocyte-like phenotype in cATMSCs [22], it did not promote the expression of cardiomyocyte-specific proteins, rhythmic calcium oscillations, or potential-dependent fluorescence emissions in these cells [34]. In the following, we also describe how UCBMSC-embedded fibrin patches could not effectively induce cardiac-specific markers, such as cTnI, in MI hearts [36, 37].

Despite the apparent divergent nature of cATMSCs and UCBMSCs, they share promising cardiovascular potential and immune regulatory capabilities. Thus, both can be immunologically safe and valuable for clinical use. However, cATMSCs are typically extracted from older donors, with intrinsic characteristics and risk factors that might lead to poor stem cell functionality. Another restriction associated with cATMSCs is that, because these cells are located in cardiac adipose tissue, they are not readily accessible. Nevertheless, cardiac fat biopsies for cATMSC isolation can be obtained easily via left lateral thoracotomy prior to coronary artery bypass surgery in patients with stable ischemia. Moreover, other procedures can be envisioned. Indeed, a large number of cardiac interventions are routinely performed in every major hospital, and each of these interventions provides an opportunity for obtaining a cardiac fat biopsy with negligible additional risk to the patient or cost to the healthcare system. Alternatively, a UCB sample can be safely, painlessly extracted for subsequent MSC isolation, and these UCBMSCs can be cryopreserved for a long time without losing regenerative and “immunoprivileged” properties. Furthermore, UCB carries a lower risk of transmitting viral infections or somatic mutations, compared to adult tissues. In the context of cell-based therapies, UCBMSCs require less culture time than cATMSCs to achieve ex-vivo expansion to a fixed number of cells; therefore, UCBMSCs are less likely to display apoptotic traits. However, both cATMSCs and UCBMSCs may be potentially expanded and banked for later allogeneic use. Nevertheless, several crucial challenges remain. First, more accurate quality and potency assays are needed to achieve UCB-based product manufacturing and to gain accreditation; second, broad agreements are needed between the international research community and blood services/UCB banks to support active collaborations and to make use of small-volume UCB units that are now discarded [30].

## Stem cell conditioning by electromechanical stimulation

Since biophysical signals to which cardiac cells are exposed constantly and specifically may also affect stem cell functions once implanted, we designed a novel ad-hoc device for supplying electrical and mechanical stimuli, individually or synchronously, to condition stem cell culture monolayers. Once developed, we examined the cardiomyogenic effects of these stimuli. For example, we tested whether they mimicked the cardiac environment, induced the maturation of trained cATMSCs [38], and promoted their integration into postinfarcted myocardium in mice [39, 40].

Remarkably, when electromechanical conditioning was applied to cATMSCs, in vitro, they displayed a cardiomyogenic-like phenotype [38, 39]. When tested in a murine MI model, conditioned cATMSCs drove the recovery of cardiac function and increased the density of myocardial vasculature [40]. In particular, at 21 days after implantation of an electromechanically conditioned cATMSC–fibrin patch, the left ventricular ejection fraction increased up to 12% in stimulated grafts, compared to untreated animals. Graft vascularization and integration with the host blood supply also resulted in increased vessel density in the infarct border region. Trained cells placed in the implanted fibrin patch also exhibited primary cardiac markers and migrated into the underlying ischemic myocardium. These studies pioneered the benefits of electromechanically stimulated cells in an in-vivo scenario. They showed that this physiological strategy held promise for stem cell training (either in cell suspension or within engineered tissue) before cell implantation to recover cardiac function post MI. In our opinion, although the question of how to produce greater numbers of trained cells remains unresolved, this technology is ready to undergo preclinical testing.

## Cardiac TE-based exploitation of cATMSCs and UCBMSCs

As mentioned previously, most efforts in the field of cardiac regeneration after MI have focused on cell therapy. Clinically, the vast majority of reported studies, mostly using bone marrow-derived mononuclear cells (revised in [41]), are intricate to compare because the delivered cells are either mixed or enriched populations, and the number of implanted cells, delivery methods, and injection time intervals are not usually comparable. Other experiences have included MSCs harvested from bone marrow aspirates [42, 43], subcutaneous adipose tissue [44], and Wharton’s jelly [45]. In general, studies have commonly employed the intracoronary or intramyocardial cell delivery routes, and use of these routes has been associated with low ratios of cell engraftment and survival. Hence, despite being safe and technologically feasible, conventional cell therapy approaches in humans have not reproduced the benefits in cardiac function restoration observed in preclinical animal models, because of the difficulties involved in repairing usually large myocardial scars and because of the low efficacy of administered cells, due to low retention rates, poor survival, and lack of differentiation. Consequently, increasingly more studies are based on techniques involving cardiac TE, which aims to combine stem cells with synthetic or natural scaffolds with characteristics very similar to those of native myocardial tissue. Once locally delivered/implanted in the infarcted area, these innovative bioactive constructs can integrate effectively into target tissues to regenerate myocardial scars and improve cardiac function [46, 47].

Regarding natural scaffolding materials for cardiac repair after MI, collagen scaffold-associated benefits have been observed in different MI models using subcutaneous adipose tissue-derived MSCs [48, 49]. In rats, MSCs from brown adipose tissue also improved cardiac function and contractility when applied into the infarcted area inside a chitosan scaffold [50]. Other in-vivo experiences have included the use of alginate, hyaluronic acid, gelatin, and matrigel (revised in [51]). Alternatively, our group has evaluated the implantation of fibrin as a scaffold material for cardiac repair (revised in [37]). Thus, we generated 3D engineered fibrin patches filled with cATMSCs or UCBMSCs and delivered them to postinfarcted myocardium in mice [36, 40, 52]. Fibrin patches filled with stem cells can be placed on top of myocardium undergoing scarring. This procedure avoids many of the drawbacks of conventional cell-infusion systems. Fibrin has several advantages; it can be extracted from the patient’s blood; it is easily readjusted; the implantation procedure is simple; it promotes viability and early proliferation in delivered cells; and it provides benefits, even when a fibrin patch does not contain cells. In our studies, new functional vascular growth and improved cardiac function were commonly observed in animals treated with fibrin–MSC patches. However, the fates of implanted cells appeared to depend on the cell type. Implanted UCBMSCs exclusively contributed to vascular growth, and implanted cATMSCs exhibited cardiac and endothelial properties. In some of these pioneering studies, we employed noninvasive bioluminescence imaging to track the behavior and survival of implanted cells [36, 52]. We found that, although fibrin patches enhanced MSC retention, their migration toward injured myocardium and survival were limited, regardless of the MSC origin. These restrictions limited the therapeutic outcomes. In addition, we had to scale up the production of fibrin–cell patches to achieve comparable or better results in humans.

Other studies have commonly employed swine as the translational model for delivering microporous membranes filled with cATMSCs after MI. In particular, in these studies, scaffolds from porcine myocardium or human pericardium have been assessed [53–57]. Succinctly, these scaffolds comprised a filamentous extracellular matrix, from which all cellular and nuclear content are removed in a process called decellularization. After decellularization, these natural scaffolds preserved fiber disposition and structure; promoted high levels of cell repopulation; closely matched the native, physiological microenvironment; preserved the inherent stiffness, composition, vasculature network, and 3-D framework of myocardium; and enabled electromechanical coupling with the host myocardium upon implantation. Moreover, once implanted in the ischemic myocardium, these engineered bioimplants improved cardiac function, reduced infarct size, attenuated fibrosis progression, and promoted both neovascularization and neoinnervation. Thus, we concluded that cardiac TE provided promising beneficial effects without any identified adverse side effects, facilitating its clinical translation.

As also mentioned previously, our laboratory was the first to describe the cardioreparative potential of the adipose tissue that surrounds the heart and pericardium, and we proposed cATMSCs as a prospective source of MSCs and a biological matrix. We also envisioned a novel approach (AGTP) for integrating cardiac adipose tissue-derived cell therapy with TE for repairing damaged myocardium. In the AGTP, a vascularized adipose flap was transpositioned over the infarcted area, which we demonstrated in the porcine MI model [58, 59]. However, recently, the risks associated with open chest surgery have brought about the advent of alternative surgical approaches; in addition, it might be beneficial to provide the AGTP to patients who do not need coronary artery bypass grafts. Consequently, we reasoned that a minimally invasive AGTP approach (mi-AGTP) would be desirable for clinical settings. Therefore, we assessed the mi-AGTP in the swine model with thoracoscopy [60]. This novel surgical technique provided beneficial effects for left ventricular function and inhibited myocardial remodeling following acute MI [58]. We then assessed the effect of postinfarction scar coverage with the AGTP in a pig model of chronic MI. There, the flap was placed on the scar 2 weeks after artery occlusion with a coil. One month after the AGTP, histopathologic analysis confirmed a reduction in infarct size and the presence of vascular connections at the flap–myocardium interface. Nevertheless, at the functional level, we did not detect significant changes in LV ejection fraction or end-systolic and end-diastolic volumes [59]. Thus, this innovative approach took advantage of local existing tissue to limit the size of the infarct scar, which simplified the surgical procedure and potentially avoided the risks associated with nonautologous cells manipulated ex vivo [61]. Thus, the AGTP intervention is expected to be readily adaptable to clinical practice; it is technically simple, it does not require additional or expensive material, and it does not incur any ethical or social concerns that could constrain its employment. Of note, we reported the first-in-man clinical trial (ClinicalTrials.gov NCT01473433, AdiFLAP Trial—AGTP-I), which investigated the safety and efficacy of the AGTP in patients with chronic MIs who underwent coronary artery bypass graft surgery [62]. Our experience demonstrated that the AGTP was safe; treated patients showed trends of smaller left ventricular end-systolic volume and smaller necrosis ratios. However, the AGTP did not completely reverse myocardial dysfunction. These encouraging results led to an ongoing 1-year follow-up multicenter randomized controlled trial (ClinicalTrials.gov NCT02798276, AGTP-II) to test AGTP efficacy. Eligible patients included candidates for surgical revascularization in one or more myocardial areas with a nonrevascularizable area. The trial was designed to validate the ability of AGTP to reduce necrotic areas [63].

## Translating MSC culture methods into good manufacturing practices for clinical use: process development technologies and regulatory issues

**Fig. 2:**
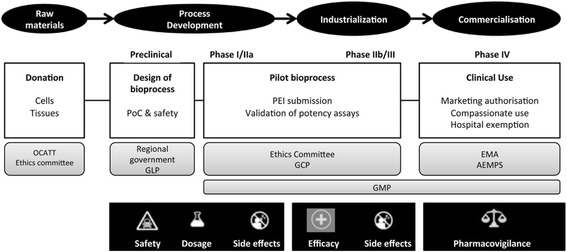
Milestones to accomplish in the development of MSC-based therapies for cardiac tissue regeneration. Donation of cells and tissue must be approved by competent authorities (OCATT) and the local ethics committee. Preclinical research and development focuses on studying the efficacy and safety (PoC) of the new, advanced therapy medicinal candidate in proof-of-concept studies; these studies must be in compliance with quality standards, when required (GLP). Once the PEI (equivalent to the Investigational Medicinal Product Dossier) is approved by the regulatory authorities, actual production of clinical-grade MSCs is performed in clean rooms, in accordance with GMP. Clinical testing is performed in controlled trials under GCP. Eventually, the authorization for marketing the MSC-based product is granted by the EMA; alternatively, the MSC-based product can be authorized for compassionate uses and nonindustrial production by the national competence authority (AEMPS), under the Hospital exemption clause. OCATT Organització Catalana de Transplantaments (Catalan), PoC proof-of-concept studies, GLP good laboratory practice, PEI Producto en Investigación (Spanish), GMP good manufacturing practice, GCP good clinical practice, EMA European Medicines Agency, AEMPS Agencia Española de Medicamentos y Productos Sanitarios (Spanish)

To date, much effort has been focused on developing allogeneic cell therapy products, such as MSC-based therapies, in accordance with current regulations [64] (Table 2). Using allogeneic versus autologous MSCs offers several advantages. Allogeneic MSCs can potentially be preserved in banks, they can be characterized comprehensively at the time of clinical use, and they cost less to produce than autologous MSCs. The large-scale production of allogeneic MSC-based products requires a variety of specialized facilities, skilled personnel, and sufficient financial resources to generate safe and effective cells consistently, from batch to batch, in compliance with current good manufacturing practice (GMP) regulations. The main milestones in the development process of MSC-based therapies for cardiac tissue regeneration are summarized schematically in Fig. 2 [65, 66]. Producing sufficient numbers of cells to support clinical trials, from first in man to phase II, is feasible in small GMP facilities, such as those found in academic institutions, hospitals, and blood banks [67]. Subsequently, complying with GMP in non-Pharma environments requires a strategy based on gradually increasing product quality. Therefore, non-Pharma environments must be capable of consistent, future production of market-approved, advanced therapy medicinal products (ATMPs), including cell and gene medicinal products, tissue-engineered products, and combined ATMPs. In addition, personnel must be trained and manufacturing processes must be optimized [68]. ATMPs that are not intended for marketing and are not industrially prepared are placed outside the standard Directive (2001/83/EC) and Regulations (726/2004). This placement is commonly called a “hospital exemption”; it is restricted to ATMPs that are “prepared on a non-routine basis, according to specific quality standards, and used within the same Member State in a hospital, under the exclusive professional responsibility of a medical practitioner, in order to comply with an individual medical prescription for a custom-made product for an individual patient” [69].

**Table 2:** Relevant guidelines and regulations in developing MSC-based medicines for treating myocardial infarction

	Guidelines and regulations
General	•European Medicines Agency Regulation (EC) No 1394/2007, Advanced Therapy Medicinal Products (amends Directive 2001/83/EC, 6 November 2001, O.J. L 311 and Regulation (EC) 726/2004, 31 March 2004, O.J. L 136). *Official Journal of the European Union* L 324. 13 November 2007
	•Directive 2001/83/EC of the European Parliament and of the Council of 6 November 2001 on the Community code relating to medicinal products for human use
	•Guideline on human cell-based medicinal products (EMEA7CHMP7410869/2006)
Preclinical	•Nonclinical safety studies for the conduct of human clinical trials and marketing authorization for pharmaceuticals (ICH Topic M3 (R2))
	•Note for guidance on preclinical safety evaluation of biotechnology-derived pharmaceuticals (CPMP/ICH/302/95)
	•Safety pharmacology studies for human pharmaceuticals (ICH Topic S7 A)
	•European Medicines Agency: 2004/9/EC—On the inspection and verification of good laboratory practice (GLP).
	•European Medicines Agency: 2004/10/EC—On the harmonization of laws, regulations, and administrative provisions relating to the application of the principles of good laboratory practice and the verification of their applications for tests on chemical substances.
Manufacturing	•Commission Delegated Regulation (EU) No 1252/2014 of 28 May 2014 supplementing Directive 2001/83/EC of the European Parliament and of the Council with regard to principles and guidelines of good manufacturing practice for active substances for medicinal products for human use
	•European Medicines Agency: Directive 2003/94/EC—Laying down the principles and guidelines of good manufacturing practice with respect to medicinal products for human use and investigational medicinal products for human use.
Clinical testing	•European Medicines Agency: Directive 2005/28/EC—On the wholesale distribution of medicinal products for human use.
	•European Medicines Agency: 2001/20/EC—On the approximation of the laws, regulations, and administrative provisions of the Member States relating to the implementation of good clinical practice in the conduct of clinical trials on medicinal products for human use.

Remarkably, a number of treatments involving MSCs are undergoing clinical testing, despite the associated challenges and costs [70, 71]. Most of these treatments rely on open/semi-open systems, which are labor intensive, require manual processing, and represent high investments in classified environments. Moreover, open/semi-open systems are associated with considerable, increasing costs, risk of contamination, variability across batches, and a lack of real-time process control. However, there has been a tendency toward developing automated platforms, including bioreactors, which may simplify the workflow and optimize resources. These platforms have increasingly impacted the robustness, traceability, and yields of clinical-grade cells; they have reduced the cost of production; and they have incorporated in-process controls that provide predictions of compliance with final product specifications [72, 73].

In developing cell media formulations, it is important to highlight the fact that animal-derived growth supplements are discouraged in the manufacture of MSCs, because they may lead to unwanted clinical effects. Most of these supplements have undergone limited characterization, and they might harbor potential animal pathogens that remain obscure. Thus, it is imperative to validate alternative media formulations suitable for large-scale expansion of cATMSCs. Although human supplements, such as human serum (HS), might have advantages over fetal bovine serum (FBS), current studies have shown that MSCs performed better in cultures supplemented with FBS rather than HS. This issue needs to be addressed early in the design of large-scale production strategies. Growth medium conditions must be devoid of substances derived from animals (xenogenic) and human products (i.e., HS and platelet lysate) and optimized with fully defined (ideally chemically defined) media formulations [70, 74]. Novel cell culture technologies and regulatory issues regarding MSC manufacturing substantially increase the overall production costs. Therefore, it is advisable to optimize MSC isolation, expansion, and storage procedures first, and then to store MSCs in master cell banks; later, the MSCs can be validated in serum-free, animal component-free reagents at the time of clinical use.

It is important to highlight that increasing GMP compliance is expected to be accomplished during the clinical development of any ATMP. In the case of master cell banks of MSC for allogeneic use, it is likely that reagents and procedures may differ between investigational and commercial manufacturing, as well as along phases of the clinical studies. In these cases, if a single master cell bank is expected to last the lifecycle of the final product, greater control over the process is required, including revalidation of master cell banks and/or the working cell bank. We typically use assays addressing product toxicity, viability, identity, purity, and potency [67, 75].

## Conclusions

The cardiovascular potential of UCBMSCs and cATMSCs has been assessed extensively in our laboratory. Our work advanced the concept that living cells seeded onto appropriately configured scaffolds could generate new tissues or organs. This approach was rapidly recognized as an alternative therapy that facilitated self-repair, reversed or attenuated adverse remodeling, and ultimately achieved long-term functional stabilization and improved heart function. The great cardiomyogenic potential exhibited by cATMSCs was also observed in large mammals, such as swine, which is the preferred preclinical model, due to its high similarity to humans. However, access to sufficient numbers of cells following standardized cell culture conditions represents a major challenge for future clinical use. Moreover, therapeutic outcomes could be limited by poor stem cell functionality, due to donor characteristics, like intrinsic pathophysiological conditions, comorbidities, and cardiovascular risk factors. Ideally, cATMSCs should be isolated with minimally invasive procedures from young donors with no cardiac pathology, no comorbidities, and no cardiovascular risk factors. Once expanded in vitro, these theoretically highly-active stem cells could be banked for later allogeneic use. In addition, in-depth studies are crucial for elucidating the immunomodulatory actions and mechanisms for the resolution of inflammation and regeneration of injured tissue by cATMSCs and UCBMSCs. These effects could be firstly mediated temporarily by paracrine mediators, but secondarily by the modulation of the host’s immune cells. On the other hand, UCBMSCs have exhibited promising vascular potential, both in vitro and in vivo, but their cardiomyogenic potential appeared to be somewhat limited. Indeed, UCBMSCs constitute a valuable model for analyzing mechanisms that govern vascular growth for tissue repair. However, in basic research, such as our TE studies, UCBMSC use has been restricted, in part, due to the lack of a broad consensus among UCB banks for collaborations, and in part due to the limited availability of small-volume UCB units, which are generally discarded.

Regarding our experiences with TE, despite the finding that fibrin patches could enhance the survival and function of implanted cells, the benefits were modest and clinical translation is currently impractical. In contrast, bioactive engineered grafts or bioprostheses can be generated by combining cATMSCs with acellular cardiac scaffolds that display preserved cardiac extracellular matrix proteins. The intrinsic structural and mechanical properties of those grafts may be better suited for restoring cardiac function post MI. However, it might not be necessary to isolate and manipulate cells ex vivo, because the AGTP constitutes a reliable step toward a new option for treating heart failure after MI. Therefore, based on our good preclinical results, we believe this is the right time for clinical translation of decellularized matrices derived from cardiac tissue biopsies. Accordingly, we are now facing complex regulatory concerns to assemble a cell product based in some of our extensively investigated MSCs or additional sources with a scaffold that is highly similar to native myocardium and practically devoid of DNA content. To that end, it is essential to meet the requirements of GMP by handling the final product strictly according to the guidelines of the Spanish Agency for Medicines and Healthcare Products.

## Abbreviations

AdiFLAP: Pericardial adipose pedicle
AEMPS: Agencia Española de Medicamentos y Productos Sanitarios (Spanish)
AGTP: Adipose graft transposition procedure
ATMP: Advanced therapy medicinal product
cATMSC: Cardiac adipose tissue-derived mesenchymal stem cell
CD31: Cluster of differentiation 31
Cx-43: Connexin 43
EMA: European Medicines Agency
FBS: Fetal bovine sera
GCP: Good clinical practice
GLP: Good laboratory practice
GMP: Good manufacturing practices
HS: Human sera
ICREC: Insuficiència Cardíaca i Regeneració Cardíaca (Catalan)
MI: Myocardial infarction
MSC: Mesenchymal stem cell
OCATT: Organització Catalana de Transplantaments (Catalan)
PEI: Producto en Investigación (Spanish)
PoC: Proof-of-concept studies
SERCA-2: Sarcoplasmic reticulum Ca^2+^ ATPase 2
Tbx-5: T-box 5
TE: Tissue engineering
UCB: Umbilical cord blood
UCBMSC: Umbilical cord blood-derived mesenchymal stem cell
β-MHC: Beta-cardiac myosin heavy chain

## References

[1] Heidenreich PA, Albert NM, Allen LA, Bluemke DA, Butler J, Fonarow GC, Ikonomidis JS, Khavjou O, Konstam MA, Maddox TM, Nichol G, Pham M, Piña IL, Trogdon JG, American Heart Association Advocacy Coordinating Committee, Council on Arteriosclerosis, Thrombosis and Vascular Biology, Council on Cardiovascular Radiology and Intervention, Council on Clinical Cardiology, Council on Epidemiology and Prevention, Stroke Council (2013). Forecasting the impact of heart failure in the United States: a policy statement from the American Heart Association. Circ Heart Fail.

[2] Roura S, Gálvez-Montón C, Lupón J, Bayes-Genis A (2017). Biotherapies and biomarkers for cardiovascular diseases: a 15-year journey at the ICREC (Heart Failure and Cardiac Regeneration) Research Laboratory in Barcelona, Spain. Eur Heart J.

[3] Laflamme MA, Murry CE (2011). Heart regeneration. Nature.

[4] Oberpriller JO, Oberpriller JC (1974). Response of the adult newt ventricle to injury. J Exp Zool.

[5] Becker RO, Chapin S, Sherry R (1974). Regeneration of the ventricular myocardium in amphibians. Nature.

[6] Poss KD, Wilson LG, Keating MT (2002). Heart regeneration in zebrafish. Science.

[7] Urbanek K, Rota M, Cascapera S, Bearzi C, Nascimbene A, De Angelis A, Hosoda T, Chimenti S, Baker M, Limana F, Nurzynska D, Torella D, Rotatori F, Rastaldo R, Musso E, Quaini F, Leri A, Kajstura J, Anversa P (2005). Cardiac stem cells possess growth factor-receptor systems that after activation regenerate the infarcted myocardium, improving ventricular function and long-term survival. Circ Res.

[8] Hierlihy AM, Seale P, Lobe CG, Rudnicki MA, Megeney LA (2002). The post-natal heart contains a myocardial stem cell population. FEBS Lett.

[9] Oh H, Bradfute SB, Gallardo TD, Nakamura T, Gaussin V, Mishina Y, Pocius J, Michael LH, Behringer RR, Garry DJ, Entman ML, Schneider MD (2003). Cardiac progenitor cells from adult myocardium: homing, differentiation, and fusion after infarction. Proc Natl Acad Sci U S A.

[10] Bergmann O, Bhardwaj RD, Bernard S, Zdunek S, Barnabé-Heider F, Walsh S, Zupicich J, Alkass K, Buchholz BA, Druid H, Jovinge S, Frisén J (2009). Evidence for cardiomyocyte renewal in humans. Science.

[11] Adler CP (1975). Relationship between deoxyribonucleic acid content and nucleoli in human heart muscle cells and estimation of cell number during cardiac growth and hyperfunction. Recent Adv Stud Cardiac Struct Metab.

[12] Hsieh PC, Segers VF, Davis ME, MacGillivray C, Gannon J, Molkentin JD, Robbins J, Lee RT (2007). Evidence from a genetic fate-mapping study that stem cells refresh adult mammalian cardiomyocytes after injury. Nat Med.

[13] Porrello ER, Mahmoud AI, Simpson E, Hill JA, Richardson JA, Olson EN, Sadek HA (2011). Transient regenerative potential of the neonatal mouse heart. Science.

[14] Laflamme MA, Myerson D, Saffitz JE, Murry CE (2002). Evidence for cardiomyocyte repopulation by extracardiac progenitors in transplanted human hearts. Circ Res.

[15] Bayes-Genis A, Salido M, Solé Ristol F, Puig M, Brossa V, Campreciós M, Corominas JM, Mariñoso ML, Baró T, Vela MC, Serrano S, Padró JM, Bayes de Luna A, Cinca J (2002). Host cell-derived cardiomyocytes in sex-mismatch cardiac allografts. Cardiovasc Res.

[16] Bayes-Genis A, Muñiz-Diaz E, Catasus L, Arilla M, Rodriguez C, Sierra J, Madoz PJ, Cinca J (2004). Cardiac chimerism in recipients of peripheral-blood and bone marrow stem cells. Eur J Heart Fail.

[17] Bayes-Genis A, Bellosillo B, de la Calle O, Salido M, Roura S, Ristol FS, Soler C, Martinez M, Espinet B, Serrano S, Bayes de Luna A, Cinca J (2005). Identification of male cardiomyocytes of extracardiac origin in the hearts of women with male progeny: male fetal cell microchimerism of the hear. J Heart Lung Transplant.

[18] Bayes-Genis A, Roura S, Prat-Vidal C, Farré J, Soler-Botija C, de Luna AB, Cinca J (2007). Chimerism and microchimerism of the human heart: evidence for cardiac regeneration. Nat Clin Pract Cardiovasc Med.

[19] Deb A, Wang S, Skelding KA, Miller D, Simper D, Caplice NM (2003). Bone marrow-derived cardiomyocytes are present in adult human heart: a study of gender-mismatched bone marrow transplantation patients. Circulation.

[20] Olivetti G, Capasso JM, Meggs LG, Sonnenblick EH, Anversa P (1991). Cellular basis of chronic ventricular remodeling after myocardial infarction in rats. Circ Res.

[21] Caplan AI (2017). Mesenchymal stem cells: time to change the name!. Stem Cells Transl Med.

[22] Bayes-Genis A, Soler-Botija C, Farré J, Sepúlveda P, Raya A, Roura S, Prat-Vidal C, Gálvez-Montón C, Montero JA, Büscher D, Izpisúa Belmonte JC (2010). Human progenitor cells derived from cardiac adipose tissue ameliorate myocardial infarction in rodents. J Mol Cell Cardiol.

[23] Cutler AJ, Limbani V, Girdlestone J, Navarrete CV (2010). Umbilical cord-derived mesenchymal stromal cells modulate monocyte function to suppress T cell proliferation. J Immunol.

[24] Melief SM, Schrama E, Brugman MH, Tiemessen MM, Hoogduijn MJ, Fibbe WE, Roelofs H (2013). Multipotent stromal cells induce human regulatory T cells through a novel pathway involving skewing of monocytes toward anti-inflammatory macrophages. Stem Cells.

[25] Spaggiari GM, Abdelrazik H, Becchetti F, Moretta L (2009). MSCs inhibit monocyte-derived DC maturation and function by selectively interfering with the generation of immature DCs: central role of MSC-derived prostaglandin E2. Blood.

[26] Monguió-Tortajada M, Roura S, Gálvez-Montón C, Pujal JM, Aran G, Sanjurjo L, Franquesa M, Sarrias MR, Bayes-Genis A, Borràs FE (2017). Nanosized UCMSC-derived extracellular vesicles but not conditioned medium exclusively inhibit the inflammatory response of stimulated T cells: implications for nanomedicine. Theranostics.

[27] Perea-Gil I, Monguió-Tortajada M, Gálvez-Montón C, Bayes-Genis A, Borràs FE, Roura S (2015). Preclinical evaluation of the immunomodulatory properties of cardiac adipose tissue progenitor cells using umbilical cord blood mesenchymal stem cells: a direct comparative study. Biomed Res Int.

[28] Roura S, Pujal JM, Bayes-Genis A (2012). Umbilical cord blood for cardiovascular cell therapy: from promise to fact. Ann N Y Acad Sci.

[29] Roura S, Pujal JM, Gálvez-Montón C, Bayes-Genis A (2015). The role and potential of umbilical cord blood in an era of new therapies: a review. Stem Cell Res Ther.

[30] Roura S, Pujal JM, Gálvez-Montón C, Bayes-Genis A (2016). Quality and exploitation of umbilical cord blood for cell therapy: are we beyond our capabilities?. Dev Dyn.

[31] Liao Y, Geyer MB, Yang AJ, Cairo MS (2011). Cord blood transplantation and stem cell regenerative potential. Exp Hematol.

[32] Lee M, Jeong SY, Ha J, Kim M, Jin HJ, Kwon SJ, Chang JW, Choi SJ, Oh W, Yang YS, Kim JS, Jeon HB (2014). Low immunogenicity of allogeneic human umbilical cord blood-derived mesenchymal stem cells in vitro and in vivo. Biochem Biophys Res Commu.

[33] Prat-Vidal C, Roura S, Farré J, Gálvez C, Llach A, Molina CE, Hove-Madsen L, Garcia J, Cinca J, Bayes-Genis A (2007). Umbilical cord blood-derived stem cells spontaneously express cardiomyogenic traits. Transplant Proc.

[34] Roura S, Farré J, Hove-Madsen L, Prat-Vidal C, Soler-Botija C, Gálvez-Montón C, Vilalta M, Bayes-Genis A (2010). Exposure to cardiomyogenic stimuli fails to transdifferentiate human umbilical cord blood-derived mesenchymal stem cells. Basic Res Cardiol.

[35] Roura S, Bagó JR, Gálvez-Montón C, Blanco J, Bayes-Genis A (2013). In vitro characterization of the molecular machinery regulating umbilical cord blood mesenchymal stem cell angiogenesis: a step towards multipotent stem cell therapy for vascular regeneration. J Stem Cell Res Ther.

[36] Roura S, Bagó JR, Soler-Botija C, Pujal JM, Gálvez-Montón C, Prat-Vidal C, Llucià-Valldeperas A, Blanco J, Bayes-Genis A (2012). Human umbilical cord blood-derived mesenchymal stem cells promote vascular growth in vivo. PLoS One.

[37] Roura S, Gálvez-Montón C, Bayes-Genis A (2017). Fibrin, the preferred scaffold for cell transplantation after myocardial infarction? An old molecule with a new life. J Tissue Eng Regen Med.

[38] Llucià-Valldeperas A, Sanchez B, Soler-Botija C, Gálvez-Montón C, Roura S, Prat-Vidal C, Perea-Gil I, Rosell-Ferrer J, Bragos R, Bayes-Genis A (2014). Physiological conditioning by electric field stimulation promotes cardiomyogenic gene expression in human cardiomyocyte progenitor cells. Stem Cell Res Ther.

[39] Llucià-Valldeperas A, Sanchez B, Soler-Botija C, Gálvez-Montón C, Prat-Vidal C, Roura S, Rosell-Ferrer J, Bragos R, Bayes-Genis A (2015). Electrical stimulation of cardiac adipose tissue-derived progenitor cells modulates cell phenotype and genetic machinery. J Tissue Eng Regen Med.

[40] Llucià-Valldeperas A, Soler-Botija C, Gálvez-Montón C, Roura S, Prat-Vidal C, Perea-Gil I, Sanchez B, Bragos R, Vunjak-Novakovic G, Bayes-Genis A (2017). Electromechanical conditioning of adult progenitor cells improves recovery of cardiac function after myocardial infarction. Stem Cells Transl Med.

[41] Roura S, Gálvez-Montón C, Bayes-Genis A (2013). The challenges for cardiac vascular precursor cell therapy: lessons from a very elusive precursor. J Vasc Res.

[42] Hare JM, Traverse JH, Henry TD, Dib N, Strumpf RK, Schulman SP, Gerstenblith G, DeMaria AN, Denktas AE, Gammon RS, Hermiller JB, Reisman MA, Schaer GL, Sherman W (2009). A randomized, double-blind, placebo-controlled, dose-escalation study of intravenous adult human mesenchymal stem cells (prochymal) after acute myocardial infarction. J Am Coll Cardiol.

[43] Hare JM, Fishman JE, Gerstenblith G, DiFede Velazquez DL, Zambrano JP, Suncion VY, Tracy M, Ghersin E, Johnston PV, Brinker JA, Breton E, Davis-Sproul J, Schulman IH, Byrnes J, Mendizabal AM, Lowery MH, Rouy D, Altman P, Wong Po Foo C, Ruiz P, Amador A, Da Silva J, McNiece IK, Heldman AW, George R, Lardo A (2012). Comparison of allogeneic vs autologous bone marrow-derived mesenchymal stem cells delivered by transendocardial injection in patients with ischemic cardiomyopathy: the POSEIDON randomized trial. JAMA.

[44] Perin EC, Sanz-Ruiz R, Sánchez PL, Lasso J, Pérez-Cano R, Alonso-Farto JC, Pérez-David E, Fernández-Santos ME, Serruys PW, Duckers HJ, Kastrup J, Chamuleau S, Zheng Y, Silva GV, Willerson JT, Fernández-Avilés F (2014). Adipose-derived regenerative cells in patients with ischemic cardiomyopathy: The PRECISE Trial. Am Heart J.

[45] Gao LR, Chen Y, Zhang NK, Yang XL, Liu HL, Wang ZG, Yan XY, Wang Y, Zhu ZM, Li TC, Wang LH, Chen HY, Chen YD, Huang CL, Qu P, Yao C, Wang B, Chen GH, Wang ZM, Xu ZY, Bai J, Lu D, Shen YH, Guo F, Liu MY, Yang Y, Ding YC, Yang Y, Tian HT, Ding QA, Li LN, Yang XC, Hu X (2015). Intracoronary infusion of Wharton’s jelly-derived mesenchymal stem cells in acute myocardial infarction: double-blind, randomized controlled trial. BMC Med.

[46] Vunjak-Novakovic G, Lui KO, Tandon N, Chien KR (2011). Bioengineering heart muscle: a paradigm for regenerative medicine. Annu Rev Biomed Eng.

[47] Hirt MN, Hansen A, Eschenhagen T (2014). Cardiac tissue engineering: state of the art. Circ Res.

[48] Araña M, Gavira JJ, Peña E, González A, Abizanda G, Cilla M, Pérez MM, Albiasu E, Aguado N, Casado M, López B, González S, Soriano M, Moreno C, Merino J, García-Verdugo JM, Díez J, Doblaré M, Pelacho B, Prosper F (2014). Epicardial delivery of collagen patches with adipose-derived stem cells in rat and minipig models of chronic myocardial infarction. Biomaterials.

[49] Shafy A, Fink T, Zachar V, Lila N, Carpentier A, Chachques JC (2013). Development of cardiac support bioprostheses for ventricular restoration and myocardial regeneration. Eur J Cardiothorac Surg.

[50] Wang H, Shi J, Wang Y, Yin Y, Wang L, Liu J, Liu Z, Duan C, Zhu P, Wang C (2014). Promotion of cardiac differentiation of brown adipose derived stem cells by chitosan hydrogel for repair after myocardial infarction. Biomaterials.

[51] Perea-Gil I, Prat-Vidal C, Bayes-Genis A (2015). In vivo experience with natural scaffolds for myocardial infarction: the times they are a-changin’. Stem Cell Res Ther.

[52] Roura S, Soler-Botija C, Bagó JR, Llucià-Valldeperas A, Férnandez MA, Gálvez-Montón C, Prat-Vidal C, Perea-Gil I, Blanco J, Bayes-Genis A (2015). Postinfarction functional recovery driven by a three-dimensional engineered fibrin patch composed of human umbilical cord blood-derived mesenchymal stem cells. Stem Cells Transl Med.

[53] Prat-Vidal C, Gálvez-Montón C, Puig-Sanvicens V, Sanchez B, Díaz-Güemes I, Bogónez-Franco P, Perea-Gil I, Casas-Solà A, Roura S, Llucià-Valldeperas A, Soler-Botija C, Sánchez-Margallo FM, Semino CE, Bragos R, Bayes-Genis A (2014). Online monitoring of myocardial bioprosthesis for cardiac repair. Int J Cardiol.

[54] Gálvez-Montón C, Bragós R, Soler-Botija C, Díaz-Güemes I, Prat-Vidal C, Crisóstomo V, Sánchez-Margallo FM, Llucià-Valldeperas A, Bogónez-Franco P, Perea-Gil I, Roura S, Bayes-Genis A (2017). Noninvasive assessment of an engineered bioactive graft in myocardial infarction: impact on cardiac function and scar healing. Stem Cells Transl Med.

[55] Perea-Gil I, Uriarte JJ, Prat-Vidal C, Gálvez-Montón C, Roura S, Llucià-Valldeperas A, Soler-Botija C, Farré R, Navajas D, Bayes-Genis A (2015). In vitro comparative study of two decellularization protocols in search of an optimal myocardial scaffold for recellularization. Am J Transl Res.

[56] Perea-Gil I, Prat-Vidal C, Gálvez-Montón C, Roura S, Llucià-Valldeperas A, Soler-Botija C, Iborra-Egea O, Díaz-Güemes I, Crisóstomo I, Sánchez-Margallo FM, Bayes-Genis A (2016). A cell-enriched engineered myocardial graft limits infarct size and improves cardiac function: pre-clinical study in the porcine myocardial infarction model. JACC Basic Translat Med.

[57] Gálvez-Montón C, Fernandez-Figueras MT, Martí M, Soler-Botija C, Roura S, Perea-Gil I, Prat-Vidal C, Llucià-Valldeperas A, Raya Á, Bayes-Genis A (2015). Neoinnervation and neovascularization of acellular pericardial-derived scaffolds in myocardial infarcts. Stem Cell Res Ther.

[58] Gálvez-Montón C, Prat-Vidal C, Roura S, Farré J, Soler-Botija C, Llucià-Valldeperas A, Díaz-Güemes I, Sánchez-Margallo FM, Arís A, Bayes-Genis A (2011). Transposition of a pericardial-derived vascular adipose flap for myocardial salvage after infarct. Cardiovasc Res.

[59] Gálvez-Montón C, Prat-Vidal C, Roura S, Soler-Botija C, Llucià-Valldeperas A, Díaz-Güemes I, Sánchez-Margallo FM, Bayes-Genis A (2013). Post-infarction scar coverage using a pericardial-derived vascular adipose flap. Pre-clinical results. Int J Cardiol.

[60] Gálvez-Montón C, Gastelurrutia P, Diaz-Güemes I, Sanchez-Margallo FM, Bayes-Genis A (2017). Minimally invasive adipose graft transposition procedure. J Cardiovasc Transl Res.

[61] Ma H, Liu J, Qian L (2016). Fat for fostering: regenerating injured heart using local adipose tissue. eBioMediPre-clinical resultscine.

[62] Bayes-Genis A, Gastelurrutia P, Cámara ML, Teis A, Lupón J, Llibre C, Zamora E, Alomar X, Ruyra X, Roura S, Revilla A, San Román JA, Gálvez-Montón C (2016). First-in-man safety and efficacy of the adipose graft transposition procedure (AGTP) in patients with a myocardial scar. EBioMedicine.

[63] Gastelurrutia P, Gálvez-Montón C, Cámara ML, Bustamante-Munguira J, García-Pavia P, Avanzas P, Jiménez-Navarro M, Pascual-Figal D, San Román A, Crespo-Leiro MG, Manito N, Núñez J, Fernández-Avilés F, Caballero A, Teis A, Lupón J, Burgada R, Martín C, Silvia J, de Teresa E, Cánovas-López S, Revilla A, Cuenca-Castillo JJ, Martínez-Climent A, Bermejo-Thomas J, Bayes-Genis A (2017). Rationale and design of a multicenter, prospective, randomized, controlled clinical trial to evaluate the efficacy of the Adipose Graft Transposition Procedure (AGTP) in patients with a myocardial scar: the AGTP II trial. BMJ Open.

[64] Guadix JA, Zugaza JL, Gálvez-Martín P (2017). Characteristics, applications and prospects of mesenchymal stem cells in cell therapy. Med Clin (Barc).

[65] Lechanteur C, Briquet A, Giet O, Delloye O, Baudoux E, Beguin Y (2016). Clinical-scale expansion of mesenchymal stromal cells: a large banking experience. J Transl Med.

[66] Torre ML, Lucarelli E, Guidi S, Ferrari M, Alessandri G, De Girolamo L, Pessina A, Ferrero I (2015). Gruppo Italiano Staminali Mesenchimali (GISM). Ex vivo expanded mesenchymal stromal cell minimal quality requirements for clinical application. Stem Cells Dev.

[67] Vives J, Oliver-Vila I, Pla A (2015). Quality compliance in the shift from cell transplantation to cell therapy in non-pharma environments. Cytotherapy.

[68] Palau R, Van Deusen AL, Carmona G (2016). 6—Compatibility of GxP with Existing Cell Therapy Quality Standards A2—Vives, Joaquim. Guide to Cell Therapy GxP.

[69] Cuende N, Boniface C, Bravery C, Forte M, Giordano R, Hildebrandt M, Izeta A, Dominici M (2014). Legal and Regulatory Affairs Committee—Europe, International Society for Cellular Therapy. The puzzling situation of hospital exemption for advanced therapy medicinal products in Europe and stakeholders’ concerns. Cytotherapy.

[70] Ikebe C, Suzuki K (2014). Mesenchymal stem cells for regenerative therapy: optimization of cell preparation protocols. Biomed Res Int.

[71] Nitkin CR, Bonfield TL (2017). Concise review: Mesenchymal stem cell therapy for pediatric disease: perspectives on success and potential improvements. Stem Cells Transl Med.

[72] Lambrechts T, Sonnaert M, Schrooten J, Luyten FP, Aerts JM, Papantoniou I (2016). Large-scale mesenchymal stem/stromal cell expansion: a visualization tool for bioprocess comparison. Tissue Eng Part B Rev.

[73] Rojewski MT, Fekete N, Baila S, Nguyen K, Fürst D, Antwiler D, Dausend J, Kreja L, Ignatius A, Sensebé L, Schrezenmeier H (2013). GMP-compliant isolation and expansion of bone marrow-derived MSCs in the closed, automated device quantum cell expansion system. Cell Transplant.

[74] Capelli C, Pedrini O, Valgardsdottir R, Da Roit F, Golay J, Introna M (2015). Clinical grade expansion of MSCs. Immunol Lett.

[75] Oliver-Vila I, Coca MI, Grau-Vorster M, Pujals-Fonts N, Caminal M, Casamayor-Genescà A, Ortega I, Reales L, Pla A, Blanco M, García J, Vives J (2016). Evaluation of a cell-banking strategy for the production of clinical grade mesenchymal stromal cells from Wharton’s jelly. Cytotherapy.

